# Crude extract of hydatid laminated layer from Echinococcus granulosus cyst attenuates mucosal intestinal damage and inflammatory responses in Dextran Sulfate Sodium induced colitis in mice

**DOI:** 10.1186/s12950-015-0063-6

**Published:** 2015-03-17

**Authors:** Imene Soufli, Ryma Toumi, Hayet Rafa, Manel Amri, Moussa Labsi, Lila Khelifi, Ferdinando Nicoletti, Chafia Touil-Boukoffa

**Affiliations:** Department of Biology, Laboratory of Cellular and Molecular Biology, University of Sciences and Technology Houari Boumediene USTHB, Algiers, Algeria; Department of Biomedical Sciences, University of Catania, Catania, Italy

**Keywords:** Inflammatory bowel disease (IBD), Experimental colitis, Nitric oxide (NO), Inducible nitric oxide synthase (iNOS), Nuclear factor-κB, Interferon (IFN)-γ, tumor necrosis factor (TNF)-α, helminth, hydatid laminated layer, Echinococcus granulosus

## Abstract

**Background:**

Inflammatory bowel disease is an immunologically mediated disease. Notably, it is less common in countries where there is a greater risk of exposure to helminths. In our study, we examined the modulatory effect of the laminated layer extracted from the cyst wall of a helminth parasite, *Echinococcus granulosus*, on dextran sulfate sodium (DSS)-induced colitis in mice.

**Methods:**

An acute colitis was induced in BALB/c mice using 2.5% w/v DSS in drinking water. The crude extract of *E. granulosus* laminated layer was injected intraperitoneally daily, starting 3 days before colitis induction. The Disease Activity Index was monitored daily, colon length and weight were measured and histological scores were evaluated. Nitric oxide (NO) and cytokine levels (interferon γ (IFN-γ), tumor necrosis factor α (TNF-α) and interleukin 10 (IL-10)) were assessed by enzyme-linked immunosorbent assay. In addition, the colonic expression of inducible nitric oxide synthase (iNOS) and nuclear factor-κB (NF-κB) was examined. Statistical analyses were performed by one-way analysis of variance and the survival rate was analyzed by the long rank test.

**Results:**

Hydatid laminated layer pretreatment significantly improved the clinical symptoms and histological scores (*** *p* < 0.01) observed during DSS-induced colitis and maintained mucus production by goblet cells. Furthermore, treatment with hydatid laminated layer caused a significant decrease in NO, IFN-γ (** *p* < 0.01) and TNF-α production (* *p* < 0.05) and an increase in IL-10 production. These results were associated with localized downregulation of iNOS and NF-κB expression.

**Conclusions:**

Our results demonstrate the potent anti-inflammatory effects of hydatid laminated layer. Furthermore, preventive treatment with the laminated layer played a beneficial role in maintaining the integrity of the intestinal mucosal barrier against DSS-induced injury.

## Background

Inflammatory bowel disease (IBD), represented mainly by ulcerative colitis and Crohn’s disease, is a chronic relapsing inflammatory condition that mainly affects the large and/or the small intestine. Its multi-factorial etiology remains poorly understood but it is widely accepted that IBD results from an uncontrolled mucosal immune response to intestinal microflora in genetically susceptible hosts [1,2]. Dysfunction of the mucosal immune response leads to infiltration of inflammatory cells into the gut mucosa and concomitant production of large amounts of proinflammatory cytokines including interleukin 1β (IL-1ß), IL-6, interferon γ (IFN-γ), tumor necrosis factor α (TNF-α) and nitric oxide (NO) [3]. The pro-inflammatory cytokines IFN-γ and TNF-α are important inducers of inducible nitric oxide synthase (iNOS) expression in macrophages, intestinal epithelial cells and other cells [4].

Many authors have reported that high expression of iNOS, a mediator of elevated NO production in inflamed colonic mucosa, is strongly associated with the mucosal lesions observed in both human IBD pathogenesis and experimental colitis [4-9]. Moreover, the expression of proinflammatory cytokines (IL-1ß, TNF-α, IL-6) and iNOS are transcriptionally regulated by the transcription factor nuclear factor (NF)-κB [4]. In addition to these immunological factors, it has been reported that environmental factors strongly influence the onset of IBD. Thus, IBD was initially described as pathologies affecting mainly the industrialized countries. Currently, these observations are no longer accurate considering the increasing occurrence of IBD in developing countries. These observations support the “hygiene hypothesis”, which states that childhood exposure to helminths reduces the risk of developing IBD [10-12]. On the basis of these observations, many studies have focused on the protective and therapeutic effects of different helminth species in experimental colitis. Indeed, infection of mice with nematode helminths including *Heligmosomoides polygyrus*, *Trichinella spiralis*, the trematode *Schistosoma mansoni* and the cestode *Hymenolepis diminuta* demonstrated a protective effect against colitis in various animal models [13-18]. Furthermore, these studies have been extended to patients with IBD. Clinical trials demonstrated that administration of eggs from the porcine whipworm, *Trichuris suis*,  to patients with IBD attenuated the excessive intestinal inflammation [19-21].

*Echinococcus granulosus* is a helminth that causes a cystic hydatid disease. Human cysts of *E. granulosus* are mainly located in the lung or the liver [22]. The cyst consists of a fluidfilled vesicle, containing two layers, a germinal layer and an outer acellular laminated layer (LL). The LL of *E. granulosus* is composed mainly of carbohydrates, proteins and lipids [23]. It appears to play two essential roles, as a mechanical barrier and an immunological barrier against the host immune defense. In fact, the survival and persistence of the parasite in the host involves evasion strategies that may be mediated by the LL-induced inhibition of NO production [24-26]. In our study, we investigated the potential protective effect of the hydatid LL of *E. granulosus* in dextran sulfate sodium (DSS)-induced experimental colitis. Here, we report that pretreatment of colitic mice with the crude extract of LL significantly reduced the severity of colitis, both macroscopically and histologically. Interestingly, this observation correlated with a significant decrease in NO production and proinflammatory cytokine (TNF-α, IFN-γ) secretion and importantly an increase in the systemic IL-10 level. Concomitantly, localized attenuation of iNOS and NF-κB expression was evident in LL-pretreated mice.

## Methods

### Mice

**Figure 1:**
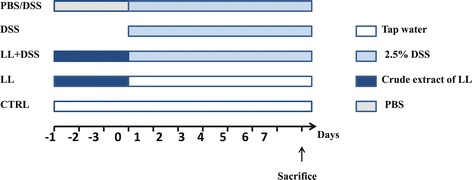
**Experimental plan.** Mice were randomly divided into five experimental groups (n = 6–10 per group). CTRL group received drinking water only. DSS group received 2.5% (w/v) DSS in their drinking water *ad libitum* for 7 days to induce acute colitis. LL/DSS group were injected daily with 200 μl LL by intra-peritoneal injection starting 3 days before and then concomitantly with DSS administration. LL group were injected daily with 200 μl of LL by intra-peritoneal injection during the 10 days. PBS/DSS group were injected daily with 200 μl of PBS by intra-peritoneal injection starting 3 days before and then concomitantly with DSS administration. The arrow shows the day when mice were euthanized.

Female BALB/c mice (8 weeks old) were purchased from the Pasteur Institute (Algiers, Algeria). These mice were acclimated for 1 week before the start of experiments and kept under normal conditions with a 12 h dark/light cycle with *ad libitum* access to food and water. Mice ranging in weight from 20–22 g were divided into four groups (n = 6–10 mice per group) as per Figure 1. This study was approved by the ethics committee of the national agency of research development in health (ANDRS).

### Initiation of acute DSS-induced colitis in mice

Mice were treated with 2.5% (w/v) DSS (MW 36 000–44 000; TdB Consultancy, Sweden) in their drinking water *ad libitum* for 7 consecutive days as described by Okayasu et al. [27] to induce acute colitis. Mice were weighed every day during the experiment and their clinical status, including weight loss, stool consistency and/or diarrhea and presence of blood in stool, was monitored. The Disease Activity Index (DAI) was determined as described previously [18,28]. Briefly, a score from 0 to 4 was given for each parameter with a maximum DAI score of 12, as follows: score 0 = no weight loss, normal stool, no blood; score 1 = 1–3% weight loss; score 2 = 3–6% weight loss, loose stool, presence of blood in stool; score 3 = 6–9% weight loss; score 4 = >9% weight loss, diarrhea, presence of fresh blood around the anus with extensive blood in the stool.

### Preparation of the crude extract of hydatid laminated layer

Hydatid cysts from human lungs were obtained from the Department of Thoracic Surgery, Mustapha Pacha Hospital (Algiers, Algeria). Each patient gave written informed consent for the study as required by the ethics committee of the ANDRS which approved the study. The crude extract of LL was prepared as described previously by Steers et al. [24]. Briefly, the hydatid cyst fluid was aseptically removed by needle aspiration. Then, the hydatid cyst membrane was washed several times in phosphate-buffered saline (PBS) supplemented with 300 UI penicillin and 0.3 mg/ml streptomycin and frozen overnight to help the removal of the LL. The latter was removed and sonicated in PBS containing a cocktail of protease inhibitors (Invitrogen, Life Technologies, Carlsbad, CA, USA), for 10 s per min on ice until a particulate solution was obtained. Endotoxin contamination was removed using endotoxin removing resin (Pierce Biotechnology, ThermoFisher Scientific, Waltham, MA, USA). The absence of lipopolysaccharide in the crude extract was verified using the Endosafe Limulus Amebocyte Lysate test (Charles River Laboratories, Wilmington, MA, USA).

BALB/c mice were injected daily with 200 μl crude extract of LL by intraperitoneal injection, starting 3 days before and then concomitantly with DSS administration.

### Plasma collection

Mice were anesthetized with chloroform, and blood was collected via cardiac puncture. Blood was centrifuged to isolate plasma, which was kept at-20°C until use.

### Preparation of peritoneal macrophages and cell culture

Macrophages were obtained by peritoneal lavage of mice using 5 ml of sterile PBS (pH = 7.4). Cellular suspensions were washed three times with sterile PBS by centrifugation at 2800 rpm for 5 min, and then the pellets re-suspended in Dulbecco’s modified Eagle’s medium (DMEM) supplemented with 10% heat inactivated fetal bovine serum, 100 U/ml penicillin, 0.1 mg/ml streptomycin and 20 mM L-glutamine. Cells were cultured at a density of 4 × 10^5^ cells/well in 24-well tissue culture plates. After 2 hours, wells were washed using sterile PBS to remove non-adherent cells. Peritoneal macrophages (pMΦ) were incubated in complete DMEM at 37°C and 5% CO_2_ for 24 h. The cells used for all experiments were cultured in triplicate. After 24 h, the supernatants were removed and stored at-20°C for nitrite concentration measurement.

### Nitrite concentration measurement

The Griess reaction was used to determine nitrite levels as an indicator of NO production, in all culture supernatants and plasma, as described previously [29]. Briefly, 100 μl of each sample was mixed with 50 μl of Griess reagent (5% sulfanilamide, 0.5% napthylethylenediamine dihydrochloride, 20% HCl). Samples were incubated at room temperature for 20 min and the absorbance read at 543 nm by spectrophotometer. The nitrite concentration was determined using a standard curve constructed with sodium nitrite (NaNO_2_; 0–200 μmol/ml).

### Measurement of systemic cytokine levels

Systemic levels of IFN-γ, TNF-α and IL-10 were determined in the plasma of mice using commercial enzyme-linked immunosorbent assay (ELISA) kits according to the manufacturer’s instructions (Invitrogen).

### Assessment of colonic damage

After euthanasia, the colon was isolated and carefully cleaned of mesentery tissue, vessels and fat, and the length measured. A 4 cm segment of the distal colon was removed and weighed after rinsing the lumen with PBS. For the histological examination, 1 cm segments from the distal colon were fixed in 10% formalin, mounted in paraffin blocks and cut into 4-μm-thick sections. The sections were stained with hematoxylin and eosin (H & E) to study histological changes and with the Alcian blue method to evaluate mucus secretion by goblet cells. Sections were assessed in a blinded fashion as previously described [18,30]. Briefly, a combined score of inflammatory cell infiltration and tissue damage was determined as follows.

- Cell infiltration: score 0, occasional inflammatory cells in the lamina propria; score 1, increased infiltrate in the lamina propria predominantly at the base of crypts; score 2, confluence of inflammatory infiltrate extending into the mucosa; score 3, transmural extension of infiltrate.
- Tissue damage: score 0, no mucosal damage; score 1, < 50% loss of crypts in large areas; score 2, ≥ 50% loss of crypts in large areas, epithelium intact; score 3, total loss of crypts in large areas and epithelium loss.

### Investigation of NF-κB and iNOS expression by immunofluorescence

An immunofluorescence study of NF-κB and iNOS expression was performed on formalin-fixed, paraffin-embedded samples. Sections of 3-μm-thick tissue were deparaffinized using xylene solvent, and rehydrated through a graded series of ethanol. The sections were then subjected to antigen retrieval in citrate buffer at pH 6.0, 95°C for 45 min. Sections were blocked for 2 h with 5% skim milk in PBS, then incubated with rabbit monoclonal antibodies against mouse NF-κB p65 subunit and iNOS (Santa Cruz Biotechnology, Dallas, TX, USA; diluted 1:100) overnight at 4°C. Finally, sections were labelled with anti-rabbit IgG-FITC (Life Technologies, diluted 1:1000) for 1 hour. Sections were washed three times with PBS between each step. Slides were covered with Kaiser’s glycerin-PBS and examined using a Zeiss inverted fluorescence microscope (Zeiss, Oberkochen, Germany). Pictures were taken using a digital camera at × 40 magnification.

### Statistical analysis

All data are expressed as the means ± standard error of the mean (SEM). Statistical analyses were performed by one-way analysis of variance to compare both time (days of DSS) and treatment effects. Probability values of *p* < 0.05 were considered to be statistically significant.

## Results

### Preventive treatment with hydatid LL ameliorates clinical symptoms of DSS-induced colitis

**Figure 2:**
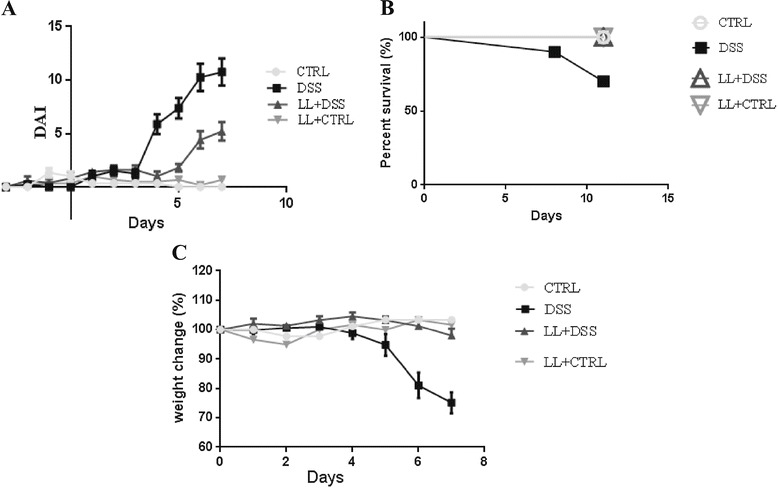
**Pretreatment with hydatid ameliorates clinical symptoms of DSS-induced colitis**. BALB/c mice were pretreated with the crude extract of LL starting 3 days before and then concomitantly with DSS administration. **A**, Weight loss, stool consistency, and fecal blood were scored to provide the DAI for each group. **B**, Survival rate of mice during the experiment. **C**, Percent change from original body weight in all groups. Data are shown for six to ten mice per group.

After administration of 2.5% DSS to mice in their drinking water, all animals developed symptoms of colitis including progressive weight loss, diarrhea and gross rectal bleeding from days 4 to 5 after the start of DSS administration. These symptoms were more marked at the end of the experimental period, when animals showed multiple clinical signs of severe disease, including marked weight loss and moribund state. Of the 10 mice in the DSS group, three died between days 5 and 7 (Figure 2). However, pretreatment with hydatid laminated layer (LL/DSS) significantly reduced the clinical score (DAI) in comparison with the DSS group. Furthermore, no moribund symptoms or mortality occurred in the LL/DSS group (Figure 2).

### Pretreatment with hydatid LL attenuates histological alterations in DSS-induced colitis

**Figure 3:**
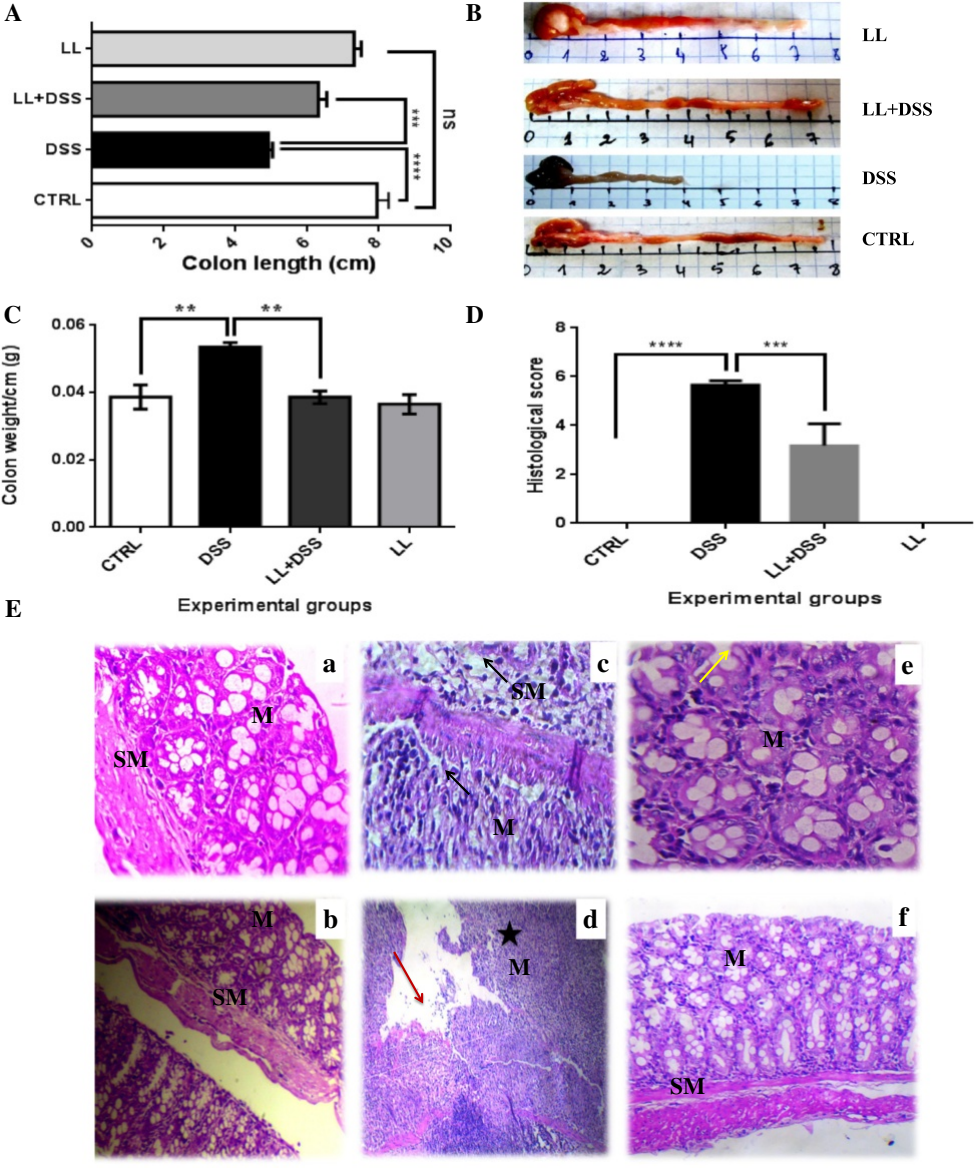
**Pretreatment with hydatid LL protects BALB/c mice from DSS-induced colitis. A** and **B**, On day 7, the colon of each mouse was removed and the length was measured; **B**, Representative photomicrographs of colons are shown. **C**, The colon of each mouse was weighed and the weight indicated is for 1 cm length of the colon. **D**, Histological scores for colon cellular infiltration and tissue alterations. All results are expressed as the mean ± standard error of mean. *p* values from LSD *post-hoc* ANOVA test are indicated (**p < 0.01, ***p < 0.001, ****p < 0.0001). The results are shown for 6 to 10 mice per group. **E**, Representative photomicrographs (×20, ×40) of H & E stained colon tissue from each group; **(a, b)** CTRL group, **(c, d)** DSS group, **(e, f)** LL + DSS group. M: Mucosa, SM: Submucosa. Red arrow: eroded mucosa, dark arrow: cell infiltrate extending throughout the mucosa and the submucosa, yellow arrow: epithelium intact, star: crypt destruction.

Shortening of the colon is used as a marker of inflammation in the DSS-induced colitis model. In the DSS-alone group the length of the colon was significantly shortened (4.6 ± 0.40 cm, **** *p* < 0.0001) compared with the control untreated group (7.95 ± 0.76 cm), whereas in the DSS/LL group the pretreatment with LL prevented the colonic shortening (6.3 ± 0.58 cm, *** *p* < 0.001, Figure 3A and B). No significant difference was observed between the LL alone group and the untreated control group (Figure 3A).

The histological analysis of the distal colon of DSS treated mice revealed marked mucosal damage, characterized by an intense inflammatory cell infiltrate extending throughout the mucosa and submucosa, crypt destruction, total depletion of goblet cells and mucosa erosion (Figure 3E). However, in the LL/DSS group, LL pretreatment significantly decreased the histological score compared with the DSS group (*** *p* <0.001, Figure 3D). The tissue alterations were also markedly reduced in LL/DSS group, with less cellular infiltration into the mucosa, more intact epithelium structure and normal crypts observed in the majority of mice of this group (Figure 3E). The untreated control group showed normal colonic mucosa and submucosa (Figure 3E) and mice from LL alone group displayed the same histological structure.

**Figure 4:**
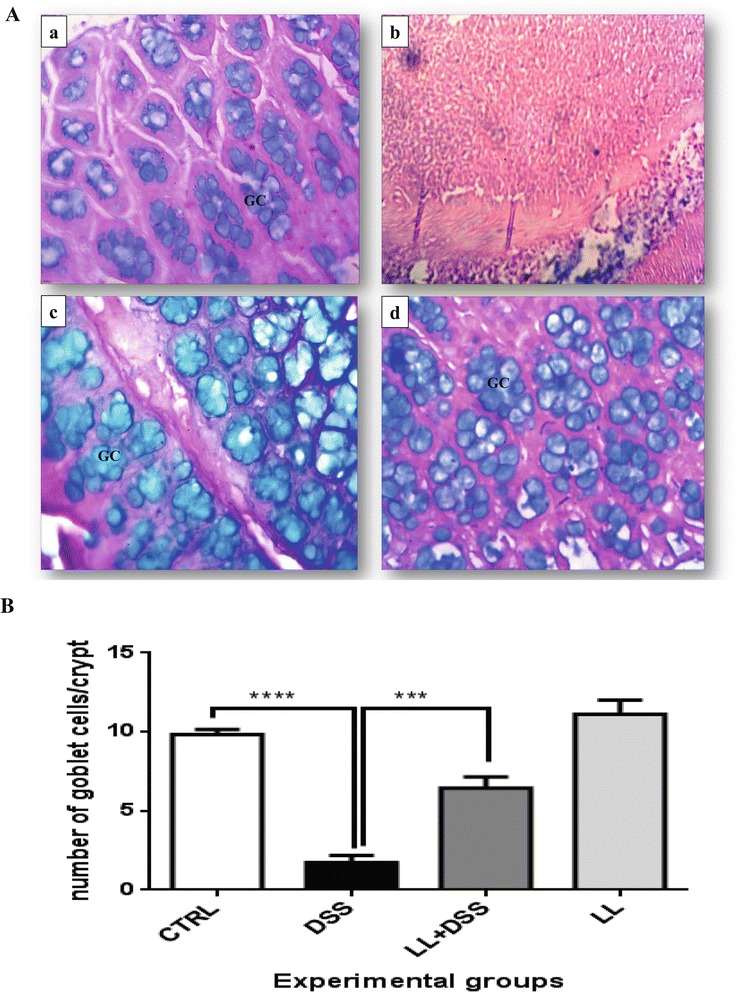
**Pretreatment with hydatid LL protects colonic goblet cells in DSS-induced colitis. A**, Representative photomicrographs of Alcian blue stained colonic tissue. **(a)** CTRL group: normal crypts and important mucus secretion, **(b)** DSS group: total depletion of goblet cells accompanied by the absence of mucus, **(c)** LL + DSS group: normal crypts and secretion of mucus by the goblet cells. **(d)** LL group: normal crypts, presence of a number of goblet cells secreting mucus. **B**, Changes in goblet cells number. All results are expressed as the mean ± standard error of the mean. *p* values from LSD post-hoc ANOVA test are indicated (***p < 0.001, ****p < 0.0001). GC: Goblet Cells.

Furthermore, histological analysis using Alcian blue staining showed enhanced mucus production in LL/DSS mice compared with the DSS group, where a total depletion of goblet cells was observed (Figure 4A). Higher numbers of goblet cells associated with mucus secretion were found in the LL/DSS group in comparison with the DSS group (*p* < 0.001, Figure 4A, B). The mice from the LL alone group displayed the same structure observed in control mice, with numerous goblet cells accompanied by mucus secretion observed in both these groups (Figure 4).

### Pretreatment with hydatid LL reduces systemic levels of NO in DSS-induced colitis

NO is an important mediator of immune and inflammatory responses. It has been shown previously that NO is highly implicated in intestinal inflammation during IBD and experimental colitis [4-9]. In our current study, we focused our attention on investigating the effect of the crude extract of hydatid LL on NO production in DSS-induced colitis in mice.

**Figure 5:**
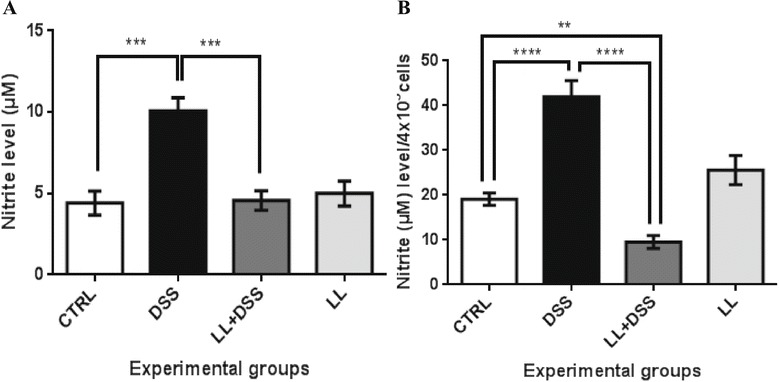
**LL pre-treatment reduces the production of nitric oxide in plasma (A) in DSS-induced colitis in BALB/c mice and in the supernatant of peritoneal macrophage cultures (B).** All results are expressed as the means ± SEM for 5 to 10 mice per group. *P* values from LSD *post-hoc* ANOVA test are indicated (***p < 0.001, ****p < 0.0001).

NO measurement in the plasma of mice showed high systemic levels of NO in mice from the DSS group compared with control group (10.07 ± 2.27 cm versus 4.40 ± 1.48 cm, *** *p* < 0.001). However, pretreatment of mice with hydatid LL caused a significant decrease in systemic NO levels in comparison with mice treated with DSS alone (*** *p* < 0.001, Figure 5A). Moreover, there was no significant difference in NO levels in plasma of mice from the LL/DSS group and the untreated control group (*p* > 0.05, Figure 5A).

### Crude extract of hydatid LL reduces nitrite level in peritoneal macrophage cultures

The nitrite level produced by cultured pMΦ was measured. Nitrite production by cultures of pMΦ obtained from colitic mice was significantly increased compared with pMΦ from untreated control mice (**** *p* < 0.0001, Figure 5B). Interestingly, NO concentration in pMΦ supernatants was lower from hydatid LL pretreated mice, compared with all other experimental groups (**** *p* < 0.0001 DSS group versus LL/DSS group, ** *p* < 0.01 LL/DSS group versus untreated control group). Conversely, animals given hydatid LL without DSS consumption showed no difference in nitrite levels in comparison with the control group (Figure 5B).

### Changes in cytokine levels *in vivo*

**Figure 6:**
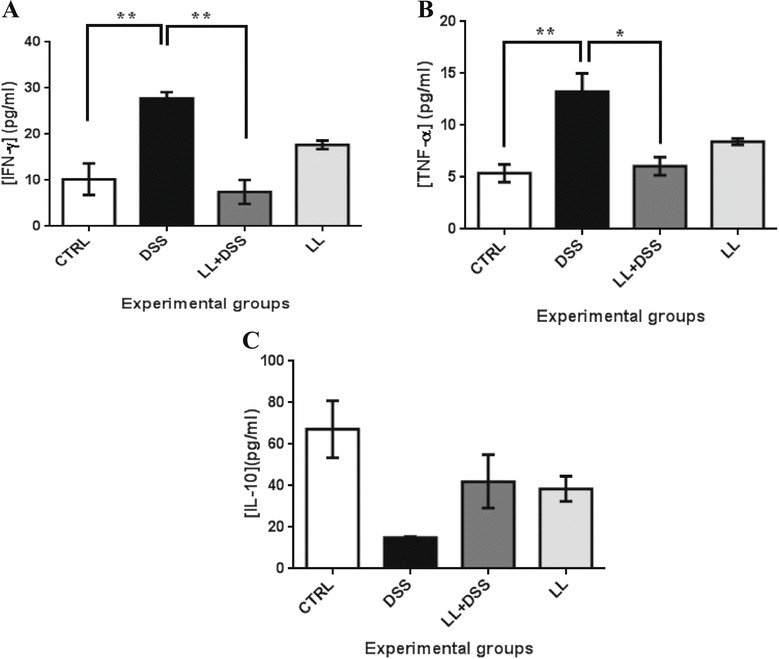
**Effect of crude extract of LL on cytokines in DSS-induced colitis.** IFN-γ (**A**), TNF-α (**B**) and IL-10 (**C**) concentration in the plasma of mice from all groups. LL-pretreatment reduces the systemic levels of IFN-γ and TNF-α and increased IL-10 systemic levels. All results are expressed as the mean ± standard error of the mean. *p* values from LSD *post-hoc* ANOVA test are indicated (*p < 0.05, **p < 0.01). *Abbreviations*: IFN-γ, interferon-γ; TNF-α, tumor necrosis factor α; IL-10, interleukin 10.

To analyze the effect of the hydatid LL on cytokine production *in vivo* during DSS-induced colitis, we measured the levels of proinflammatory cytokines (TNF-α, IFN-γ) and the immunoregulatory cytokine IL-10 by ELISA in plasma from mice (Figure 6). Systemic IFN-γ and TNF-α levels were markedly increased in the DSS group in comparison with controls (*p* < 0.01). Hydatid LL pretreatment significantly attenuated both IFN-γ and TNF-α expression compared with mice treated with DSS alone (** *p* < 0.01, * *p* < 0.05 respectively). However, treatment of control mice with LL alone also induced an increase in plasma IFN-γ and TNF-α level, although without any significant effect on overall health compared with the control group (Figure 6). The systemic levels of IL-10 were increased in the LL/DSS group in comparison with the DSS group, although no statistically significant differences were observed among the groups (Figure 6C).

### Down-regulation of iNOS and NF-κB expression in colonic tissue of mice pretreated with crude extract of hydatid LL

**Figure 7:**
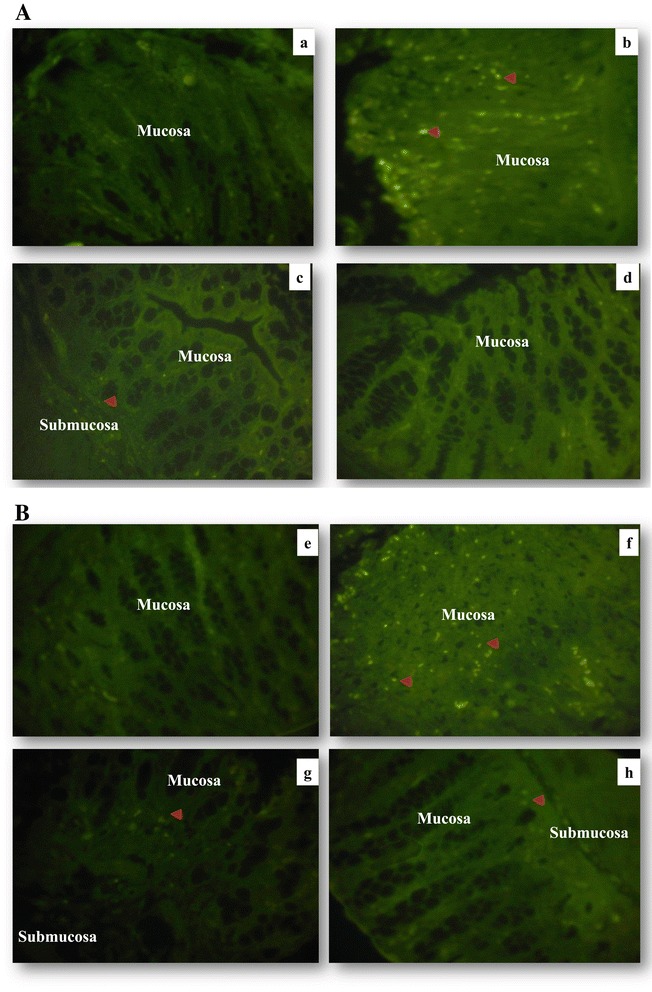
**Representative images of immunofluorescence staining**. Distal colon tissues from mice were stained with anti-iNOS (**a–d**) and anti p65 NF-κB subunit (**e–h**). Colonic tissue stained with anti-iNOS **(a)** CTRL group**, (b)** DSS group, **(c)** LL/DSS group**, (d)** LL group. Colonic tissue stained with anti-p65 subunit NF-κB **(e)** CTRL group, **(f)** DSS group, **(g)** LL/DSS group, **(h)** LL group. Red arrow indicates iNOS or NF-κB staining, all magnifications are × 40.

High expression of iNOS is considered the most important source of elevated nitrite levels in experimental colitis and in patients with IBD [4-7]. The expression of iNOS is transcriptionally regulated by NF-κB. Therefore, we examined the effect of crude extract of hydatid LL on iNOS and NF-κB expression in DSS-induced colitis in mice. Colon tissue sections were tested for the expression of iNOS and NF-κB by immunofluorescence using anti-iNOS and anti-NF-κB p65 subunit antibodies. The expression of both NF-κB and iNOS was markedly increased in the colonic mucosa of DSS treated mice in comparison with the untreated control group. This increase in expression in colonic mucosa was significantly attenuated by LL treatment (Figure 7).

## Discussion

IBD is characterized by disruption of the intestinal microbiota that leads to an excessive inflammatory response in the gut. Currently, modulation of intestinal inflammation using helminths is being explored as a promising prophylactic and therapeutic approach for IBD. Several experimental studies using different animal models of colitis have shown the ability of parasitic worms to attenuate intestinal inflammation. Furthermore, intestinal parasitic worms such as *T. suis* have been shown to have a therapeutic effect in patients with ulcerative colitis and Crohn’s disease [19-21]. The data presented here demonstrate that the pretreatment of mice with hydatid LL extracted from the cyst of the cestode parasite *E. granulosus* attenuates inflammation in a DSS-induced colitis model in mice.

The oral administration of 2.5% DSS in mice causes acute colitis. Animals from this group showed a variety of clinical symptoms of IBD including diarrhea, bloody stool and weight loss. A Disease Activity Index score was used as a reliable tool to assess the extent of the gastrointestinal disease in the DSS-induced experimental colitis. At the end of the experimental period, the clinical symptoms were more pronounced. Our observations are in agreement with those reported in other studies using the DSS-induced colitis model [27,28]. However, pretreatment of mice with hydatid LL significantly improved the clinical score, suggesting a protective effect of hydatid LL in this model of colitis. The histological scores indicate that pretreatment of mice with LL prevented mucosal damage characterized by loss of crypt glands and epithelium destruction and inhibited colon shortening as observed in the DSS-alone group. Moreover, a histological study using Alcian blue staining showed regular mucosal structure with a number of goblet cells secreting mucus in the LL/DSS group, in stark contrast to the DSS treated mice. This observation suggests that LL pretreatment may protect mice from DSS-induced colitis by promoting mucus secretion.

IBD and DSS induced colitis are characterized by elevated levels of proinflammatory cytokines including IFN-γ, TNF-α and IL-6, and also increased NO production [7,8], which contribute to the destruction of the epithelium, ulceration of the intestinal mucosa, erosion and infiltration of inflammatory cells [27,30,31]. We focused our attention on the effect of LL pretreatment on NO production and TNF-α, IFN-γ secretion knowing that these cytokines are potent inducers of iNOS expression [4,32]. Our data demonstrate an increase in NO levels both in plasma and macrophage culture supernatants of mice from the DSS group. Our results are in line with previous studies showing that intestinal inflammation observed in IBD and DSS-induced colitis is associated with prolonged iNOS activation and increased NO production [4,6,32]. In fact, NO contributes to the perpetuation of inflammation, causing increased tissue damage [33].

Many authors have reported the synergistic effect of IFN-γ/TNF-α on iNOS induction through NF-κB activation [34,35]. Notably, NF-κB activity is enhanced in the intestinal mucosa of both patients with IBD and the DSS-induced colitis model [4,36]. In our study, we reported that an elevated level of NO was associated with an increased systemic level of IFN-γ and TNF-α, and over expression of iNOS and NF-κB in colonic tissue. Interestingly, the LL/DSS group showed a significant decrease in NO production, in both plasma and pMΦ culture supernatants, compared with mice from the DSS group. Moreover, pretreatment of mice with hydatid LL induced a concomitant decrease of IFN-γ and TNF-α levels in plasma and the attenuation of iNOS and NF-κB expression in the mucosa. Moreover, other studies have described an important immunomodulatory role for hydatid LL through the downregulation of NO production in mice *in vivo* and *in vitro* [24,25].

However, administration of hydatid LL alone to BALB/c mice also induced an increase in plasma TNF-α and IFN-γ levels, although without any significant effect on mouse health. This result is probably related to the heterogeneous mix of compounds present in the crude extract of the hydatid LL, which may induce immune responses in healthy mice. Hence, identification and purification of the bioactive molecule (s) should provide more potent and effective anti-inflammatory effects. Therefore, our future aim will be to characterize the bioactive molecules within the LL extracted from the cyst wall of *E. granulosus*.

The hydatid LL is a crucial element of the host-parasite interface. It represents an important barrier against immunological host responses [26,37]. To survive, the parasite impairs Th1 protective responses and induces Th2 and regulatory T cell (Treg) responses, which are then involved in the mechanism of the immune evasion strategy. This effect may be mediated by LL antigens [26,37]. Many reports suggest the ability of helminths to down-regulate the proinflammatory immune response via the induction of Tregs or the production of antiinflammatory cytokines, for example IL-10 and tumor growth factor β, which can regulate both Th1 and Th2 responses [38]. Thus, the decrease of NO and proinflammatory cytokine levels in plasma of pretreated mice LL is likely related to the immunomodulatory effect of the cystic extract. It appears that this effect may be mediated by the immunoregulatory action of IL-10, as this cytokine was highly increased during treatment with the cystic extract (Figure 6). It has been reported that IL-10 inhibits proinflammatory cytokine production, including IFN-γ, IL-1ß, TNF-α and IL-12, leading to the inhibition of iNOS expression. It has also been suggested that IL-10 may exert its regulatory functions through NF-κB inhibition [36].

## Conclusions

Collectively, the results obtained are in favor of the hygiene hypothesis and suggest the existence of an immunoprotective effect mediated by helminths against colitis. In our model, this effect appears to be related to the action of larval structures during echinococcosis evolution. This beneficial effect of the hydatid LL could represent a promising therapeutic approach. However, further investigations are required to fully explore the safety aspects and elucidate the exact mechanism of action.

## Abbreviations

ANDRS: National agency for research and health development
CTRL: Control
DAI: Disease Activity Index
DMEM: Dubbelco’s modified Eagle medium
DSS: Dextran sulfate sodium
ELISA: Enzyme-linked immunosorbent assay
FITC: Fluorescein isothiocyante
H&E: Hematoxylin and eosin
IBD: Inflammatory bowel disease
IL: Interleukin
IFN-γ: Interferon-γ
i.p.: Intraperitoneally
LL: Laminated layer
NO: Nitric oxide
iNOS: Inducible nitric oxide synthase
NF-κB: Nuclear factor kappa B
PBS: Phosphate-buffered saline
pMΦ: Peritoneal macrophages
SEM: Standard error of the mean
TNF-α: Tumor necrosis factor α
